# Stand Up to Excite the Spine: Neuromuscular, Autonomic, and Cardiometabolic Responses During Motor Imagery in Standing vs. Sitting Posture

**DOI:** 10.3389/fphys.2021.762452

**Published:** 2021-11-23

**Authors:** Sidney Grosprêtre, Uros Marusic, Philippe Gimenez, Gael Ennequin, Laurent Mourot, Laurie Isacco

**Affiliations:** ^1^EA4660-C3S Laboratory - Culture, Sports, Health and Society, University Bourgogne Franche-Comté, Besançon, France; ^2^Institute for Kinesiology Research, Science and Research Centre of Koper, Koper, Slovenia; ^3^Department of Health Sciences, Alma Mater Europaea–ECM, Maribor, Slovenia; ^4^Université Clermont Auvergne, CRNH, AME2P, Clermont-Ferrand, France; ^5^EA3920-Prognostic Markers and Regulatory Factors of Heart and Vascular Diseases, and Exercise Performance, Health, Innovation Platform, University Bourgogne Franche-Comté, Besançon, France; ^6^National Research Tomsk Polytechnic University, Tomsk, Russia

**Keywords:** heart rate, V̇O_2_, Center of Pressure (COP), H-reflex, electromyography

## Abstract

Motor imagery (MI) for health and performance strategies has gained interest in recent decades. Nevertheless, there are still no studies that have comprehensively investigated the physiological responses during MI, and no one questions the influence of low-level contraction on these responses. Thus, the aim of the present study was to investigate the neuromuscular, autonomic nervous system (ANS), and cardiometabolic changes associated with an acute bout of MI practice in sitting and standing condition. Twelve young healthy males (26.3 ± 4.4 years) participated in two experimental sessions (control vs. MI) consisting of two postural conditions (sitting vs. standing). ANS, hemodynamic and respiratory parameters, body sway parameters, and electromyography activity were continuously recorded, while neuromuscular parameters were recorded on the right triceps surae muscles before and after performing the postural conditions. While MI showed no effect on ANS, the standing posture increased the indices of sympathetic system activity and decreased those of the parasympathetic system (*p* < 0.05). Moreover, MI during standing induced greater spinal excitability compared to sitting posture (*p* < 0.05), which was accompanied with greater oxygen consumption, energy expenditure, ventilation, and lower cardiac output (*p* < 0.05). Asking individuals to perform MI of an isometric contraction while standing allows them to mentally focus on the motor command, not challenge balance, and produce specific cardiometabolic responses. Therefore, these results provide further evidence of posture and MI-related modulation of spinal excitability with additional autonomic and cardiometabolic responses in healthy young men.

## Introduction

Motor function in sport performance or rehabilitation could be improved by mental training. The most popular modality of mental training is motor imagery (MI), the mental simulation of an action without the corresponding motor output (Decety, 1996). Numerous previous studies have shown that motor brain areas are activated during MI, such as the parietal, premotor, and primary motor cortices (Decety et al., 1994; Lotze et al., 1999; Grèzes and Decety, 2001; Ehrsson et al., 2003; Guillot et al., 2009; Munzert et al., 2009; Kilintari et al., 2016). Some recent evidence highlighted that this minor activation of motor regions possibly generates a sub-threshold brain output, which could reach spinal levels. However, at the spinal level, the general picture of MI activation is less clear, with some authors showing upward modulation of spinal excitability (Bonnet et al., 1997; Hale et al., 2003; Cowley et al., 2008; Aoyama and Kaneko, 2011; Grosprêtre et al., 2016) and others showing downward or no modulation (Oishi et al., 1994; Yahagi et al., 1996; Mouthon et al., 2015). In fact, it has been suggested that such sub-threshold cortical output was susceptible to partially reach the spinal networks by affecting the most sensitive structures, i.e., the spinal interneurons (Grosprêtre et al., 2014). More particularly, the spinal pre-synaptic inhibitory processes, mediated by primary afferent depolarizing interneurons, were shown to be decreased during MI as compared to rest (Grosprêtre et al., 2016). The lack of modulation of other larger spinal structures, such as the motoneurons themselves, may explain the absence of global spinal excitability changes observed in some previous studies. However, it was shown that repetition of this minor stimulus, such as after an acute bout of mental practice, may increase the sensitivity of all spinal structures (Grosprêtre et al., 2019).

While activation of the voluntary motor system is no longer discussed during MI, this may not be the only physiological tract triggered. Indeed, some authors pointed out a relationship between MI (i.e., human cognitive abilities) and the autonomic nervous system (ANS) (i.e., vital function regulation via sympathetic and parasympathetic activity) (Decety et al., 1991, 1993; Wang and Morgan, 1992; Fusi et al., 2005; Di Rienzo et al., 2012; Collet et al., 2013). The ANS anticipates the action in preparing cardiorespiratory responses and providing metabolic resources necessary for the motor control (Collet et al., 2013). In addition, ANS is not only activated in response to an energy demand but also to cognitive and/or emotional tasks (Collet et al., 2013). MI generates a motor preparation phase and shares part of the mechanisms underlying this motor preparation and execution. MI is thus likely to elicit changes in ANS activity (e.g., sympathetic activation) to insure the necessary cardiorespiratory responses to the upcoming expected energy expenditure (EE). Some studies have reported respiratory and hemodynamic modifications in response to mental simulation exercise (Decety et al., 1991, 1993; Wang and Morgan, 1992; Fusi et al., 2005). Interestingly, Decety et al. (1991) also noted that ventilation, during imaged locomotion at increasing speed (i.e., running), was more important than the actual metabolic demand. One hypothesis to explain this observation could be a dissociation between the appropriate ANS response in anticipation of the motor action (and thus the energy mobilization and cognitive demand required to provide this expected movement), and the lower EE induced by mental imagery *per se*. However, despite increasing interest in MI for health and performance strategies during the past decades, studies that have comprehensively investigated neuromuscular, ANS, and cardiometabolic responses during MI remain rare or non-existent.

The most common method of activating these functions at a low level and making the participant cognitively available for a MI task is to get him into a posture that requires automated low-level contractions. To this aim, performing MI in standing position allows to observe its effect with a low-grade background electromyographic (EMG) activity. Furthermore, spinal excitability of the triceps surae muscle is known to decrease from sitting or lying to standing posture (Katz et al., 1988; Koceja et al., 1995; Chalmers and Knutzen, 2002; Kawashima et al., 2003), independent of the level of background EMG (Cattagni et al., 2014). Interestingly, pre-synaptic inhibitory mechanism has been suggested as one of the main contributors to this particularly lower spinal excitability in standing as compared to sitting (Baudry and Duchateau, 2012; Johannsson et al., 2015). Then, given the pre-activation of pre-synaptic circuitry in standing position, it can be hypothesized that spinal excitability can be increased to a greater extent when practicing MI in standing posture. Regarding ANS and cardiometabolic modulations from sitting to standing, while increased sympathetic and decreased parasympathetic activity associated with greater cardiorespiratory response are often observed, EE and, overall, metabolic adjustments are not consistently reported (Miles-Chan et al., 2013; Miles-Chan and Dulloo, 2017; Amaro-Gahete et al., 2019). However, it has been shown that the metabolic changes associated with the standing posture vary widely between individuals, indicating that this position is highly sensitive to slight mild perturbations, in contrast to the more stable seated position. Therefore, the standing posture may represent a condition in which neuromuscular and metabolic factors are more prone to MI-induced modifications.

Thus, the aim of the present study was to investigate the neuromuscular, ANS, and cardiometabolic changes associated with acute bout of MI practice in sitting and standing condition. It has been shown that acute MI practice in sitting induces spinal plasticity (Grosprêtre et al., 2019). Therefore, given the pre-activation of pre-synaptic circuitry in standing, it can be hypothesized that spinal excitability is increased to a greater extent when MI is practiced in standing posture. Finally, with regards to ANS and cardiometabolic modifications, the few previous and controversial results of the literature do not allow to clearly predict an effect of MI. It could nevertheless be hypothesized that standing posture, due to its greater sensitivity, would be more subjected to ANS and cardiometabolic MI-induced modulations, as compared to sitting posture. It is now well-recognized that motor function and learning could be improved by MI in sport and health context. The present study may thus help in deciphering conditions (e.g., MI during standing) that potentiate spinal excitability and cardiometabolic responses for both athlete and patient practice.

## Materials and Methods

### Participants

Twelve healthy, moderately active young males participated in the present study (age: 26.3 ± 4.4 years, height: 1.77 ± 0.05 m, weight: 75.0 ± 10.1 kg, between 150 and 200 min of moderate to vigorous physical activity by week estimated by interview based on the Global Physical Activity Questionnaire items) and were screened before examination. Our sample size calculation is based on the primary efficacy outcome that relates to changes in spinal excitability before and after an MI session. In previous studies, an improvement of H-reflex of +15 to +35% was found during MI in a sitting position with 9–13 participants (Grosprêtre et al., 2018, 2019; Bouguetoch et al., 2020). Considering a significance level of 5%, a power of 90%, 12 participants are included to meet the objectives of the study. The sample size calculation was performed on PASS 13 Power Analysis and Sample Size Software (Machin et al., 2008). The exclusion criteria included: former or current smoker, current medication use, and the presence of obvious neurological, cardiovascular, or metabolic disease. Participants completed the revised version of the Movement Imagery Questionnaire (MIQ-r) to determine their MI ability (Hall and Martin, 1997). Participants reported an average MIQ-R score of 47.2 ± 6.7 over 56, indicating good imagery capacity. After explaining all risks and benefits associated with the study, all participants gave their written informed consent to participate in the present study. They committed to no unusual training or exercise program throughout the duration of the study, were asked to avoid any intense physical activity 48 h prior to each experimental session, and to maintain usual dietary and sleep habits throughout the duration of the study. This study protocol was approved by the institutional review board prior to participant recruitment (CPP: 2016-A00511-50) and was conducted in accordance with international ethical standards (Harriss et al., 2017) and the guidelines of the World Medical Association Declaration of Helsinki.

The rationale for our sample size was based on previous results on H-reflex modulation after MI training (Grosprêtre et al., 2018, 2019). Considering a significance level of 5% and a power of 80%, a minimum sample of 10 participants was required to meet the objectives of the study. Then, Hoffman et al. (2018) previously reported a sexual dimorphism in the H-reflex that likely underlies neurological differences between men and women. In addition, due to the difficulty of standardizing hormonal status in women and considering the potential effects of endogenous and exogenous sex hormones on spinal excitability, ANS, and metabolism, we decided to include only men (Abhishekh et al., 2013; Casey et al., 2016).

### Experimental Design

**FIGURE 1:**
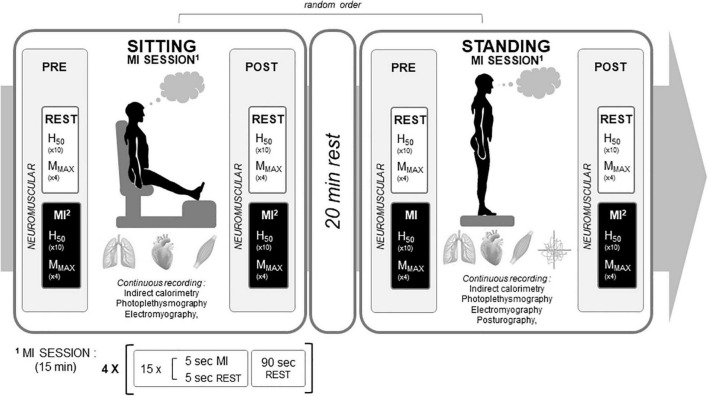
Experimental design. An experimental motor imagery (MI) condition is depicted. The control condition had the same rationale with 15 min rest observed instead of the MI training session. H_50_: H-reflex recorded at 50% of maximal H-wave amplitude. M_MAX_: maximal muscle compound action potential.

The study was conducted in two experimental sessions of about 2 h 30 min in the post-prandial state (i.e., ∼2 h after a standardized breakfast consisting of bread, butter, jam, yogurt, fruits and water; energy content represented 9.5–10 kcal⋅kg^–1^ of body mass), a MI, and control (CTRL) conditions, performed in random order. A 48-h washout period between experimental sessions was used to eliminate potential carryover effects. Each experimental session took place at the laboratory in a temperature, pressure, and humidity-controlled and quiet room (20°C, 765 mmHg, 50% of relative humidity, respectively). Each experimental session was composed of two conditions assessed randomly, one in sitting and one in standing position, interspaced by 20 min of rest (Figure 1). For the sitting condition, participants sat in a comfortable chair with the hip at 120° and the leg extended to provide a similar configuration of calf muscles from the standing position: knee at 180° and ankle at 90°. For the standing position, participants stood in bipodal upright posture with feet spaced in a natural position (shoulder width), reproduced between sessions. In both sitting and standing, some parameters were recorded PRE and POST MI and CTRL conditions (mainly evoked responses to nerve stimulation), while other parameters were continuously recorded during the experimental conditions (beat by beat hemodynamic, respiratory parameters, and myoelectrical activities). In the standing position, displacements of the Center of Pressure (CoP) were also continuously recorded during MI and CTRL conditions. Neuromuscular parameters were recorded on the right triceps surae muscles, being one of the most solicited muscle to maintain the standing posture.

After skin preparation and electrode positioning, the optimal stimulation parameters for H-reflex and M-wave recordings were determined in each experimental session. PRE neuromuscular assessment was always performed first, before the participant was equipped for continuous measurements. This sequence was followed to ensure (i) a minimum time with the mask for the assessment of gas exchanges and (ii) sufficient time after the last nerve stimulation to allow beat by beat, hemodynamic, and respiratory measurements to be taken with as little anxiety as possible about the discomfort associated with electrical stimulation. For POST measurement, neuromuscular assessments were performed immediately after the end of the CTRL or MI condition. The participants removed the facemask before the first nerve stimulation.

In the MI condition, independently of the posture, participants had to imagine a maximal isometric contraction of the right calves and to feel the sensation corresponding to this effort (kinaesthetic imagery) both during PRE and POST tests and during the 15-min sitting or standing MI training session. The MI training session in sitting and standing position consisted of four series of 15 MI trials of 5 s (5 s rest in-between), interspaced by 90 s rest between series. After each series, participants were asked to estimate their MI quality through a quotation on a Likert scale, from 1 (poor) to 7 (excellent). To avoid the dependency of postural sways on visual feedbacks, participants were asked to perform MI with their eyes open in both the sitting and standing posture. For PRE and POST tests, as well as during the training sessions, every 5 s imagined trial was preceded and ended by an auditory signal. In PRE and POST tests, during each 5 s MI trial, an electrical stimulation was delivered to elicit either H or M-waves (see below) at a random interval after the auditory signal to avoid from anticipatory mechanisms.

The CTRL condition was designed to assess the time-effect of 15 min of sustained standing or sitting posture on neuromuscular, ANS, and cardiometabolic parameters. Participants were asked to stay relaxed without thinking of any movement or activity. The EMG signals were monitored during the whole period to ensure that no minor contraction was induced during this resting period.

### Anthropometric and Body Composition Parameters

After the participant voided their bladder, body mass (kg) was assessed to the nearest 0.1 kg using calibrated scale (Digital scale Seca model 873 Omega, Germany) and standing height (m) was determined to the nearest 0.01 m using a standing stadiometer (Seca model 720, Hamburg, Germany). The weight and height of participants were measured barefoot while wearing underwear, and body mass index (BMI) was thus calculated as body weight (kg) divided by height squared (m^2^). Waist and hip circumferences (WC and HC) were measured in triplicate to the nearest 0.5 cm in a standing position with a standard non-elastic tape applied horizontally midway between the last rib and the superior iliac crest for the WC, and at the widest portion of the buttocks for the HC. The waist to hip ratio (WHR) was calculated as WC divided by HC. Body composition (fat mass and fat-free mass) and hydration were assessed using the bioelectrical impedance Tanita MC-780 (Tanita Corporation, Tokyo, Japan).

### Cardiometabolic and Autonomic Nervous System Parameters

#### Respiratory Measurements and Metabolic Assessment

After calibration following the recommendation of the manufacturer and prior each session, oxygen consumption (V̇O_2_), carbon dioxide production (V̇CO_2_), ventilation (V̇E), tidal volume (Vt), and breathing frequency (Bf) were recorded breath by breath through gas exchange measurement (MetaMax^®^, Cortex Biophysik, Leipzig, Germany). Respiratory quotient (RQ) was calculated as V̇CO_2_/V̇O_2_ and EE (kcal⋅min^–1^) as V̇O_2_ × energy equivalent of oxygen as already described (Isacco et al., 2016).

#### Autonomic Nervous System (Heart Rate Variability, Cardiac Baroreflex Sensitivity, and Hemodynamic Assessments)

Inter-beat interval derived from ECG performed at 1,000 Hz (IBI; BioAmp, ADInstruments, Sydney, NSW, Australia) and beat-to-beat blood pressure (Human NIBP Nano ADInstruments, Sydney, NSW, Australia) were measured continuously. The Human NIBP Nano measures arterial pressure using photoplethysmography of the middle phalanx of the middle finger (Wesseling et al., 1995; Imholz et al., 1998). Arterial pulse pressure (PP, mmHg) was calculated from systolic arterial pressure (SAP) minus diastolic arterial pressure (DAP). The arterial pressure signal was analyzed using LabChart 8 Pro (ADInstruments, Sydney, NSW, Australia). Stroke volume (SV) was estimated using the Windkessel Model (3-Element) (Bogert and Van Lieshout, 2005) and cardiac output (CO) was calculated as the product of HR and SV, while total peripheral resistance (TPR) was determined by dividing mean arterial pressure [MAP; (SAP+2DAP)/3] by CO.

### Neuromuscular Recordings

#### Electromyographic Activity

Electromyographic activity was recorded from five muscles of the right leg [soleus (SOL), gastrocnemius medialis (GM), gastrocnemius lateralis (GL), tibialis anterior (TA), and vastus lateralis (VL)]. The skin was first shaved and dry-cleaned with alcohol to keep low impedance (<5 kΩ). Wireless sensors equipped with two silver chloride recording points and its own reference (Delsys, Natick, MA, United States) were used to record EMG signals and firmly strapped to the leg. To record SOL EMG, the sensor was placed 2 cm below the insertion of the gastrocnemii muscles on the Achilles’ tendon. GM and GL sensors were placed over the muscle belly in line with their insertion. TA and VL EMG activity were recorded to CTRL synergists and antagonist activities in order to maximize the isolation of experimental measures (force, evoked potentials) on the triceps surae. TA EMG was recorded by placing the sensor at 1/3 of the distance on the line between the fibula and the tip of the medial malleolus, and VL at 2/3 on the line from the anterior spina iliaca superior to the lateral side of the patella. EMG signals were amplified with a bandwidth of 15 Hz to 1 kHz (gain: 1,000) and digitized on-line (sampling frequency: 2 kHz) using LabChart software (LabChart 8, ADInstruments, Sydney, NSW, Australia).

#### Peripheral Nerve Stimulation

Neuromuscular parameters were assessed by means of recording triceps surae electrophysiological responses evoked by posterior tibial nerve stimulation, such as H-reflexes and M-waves (Rozand et al., 2015). Single rectangular pulses (1-ms width) were delivered by a constant-current stimulator (Digitimer, model DS7A, Hertfordshire, United Kingdom) through self-adhesive anode (8-mm diameter, Ag-AgCL) firmly strapped to the knee in the popliteal fossa (Cattagni et al., 2018). The cathode (5 × 10 cm, Medicompex SA, Ecublens, Switzerland) was placed over the patella. The monitoring of TA EMG activity during the setting of the stimulation electrode ensured that the common peroneal nerve was not activated.

The intensity of the stimulation was progressively increased from SOL, GM, and GL response threshold with 2 mA increment until maximal H-reflex (H_MAX_) could be obtained. Since H_MAX_ can be affected by a ceiling effect that could hide potential spinal modulations induced by MI, a submaximal H-reflex was recorded in the present study (Grosprêtre et al., 2019). Subsequently, the intensity that provides an H-reflex response of 50% of its maximal amplitude (H_50_) in the ascending part of the recruitment curve was determined. The intensity was then increased with 5 mA increment until M-wave of the three muscles no longer increased. This last stimulation-intensity was increased by 20% to ensure supramaximal stimulation and used to record maximal M-wave (M_MAX_). In each condition (MI and REST) and time point (PRE and POST), four stimulations were performed to record H_MAX_ and M_MAX_, and twelve stimulations were performed to record H_50_.

### Data Analyses

#### Respiratory and Metabolic Analyses

During the MI training sessions, V̇O_2_, V̇CO_2_, EE, RQ, V̇E, Vt, and Bf values were averaged over the whole sessions, each series (4 × 2 min30), and each resting period (3 × 1 min30). Similarly, CTRL conditions were split into seven parts, reproducing times of MI training sessions and the corresponding averaged V̇O_2_, V̇CO2, EE, RQ, V̇E, Vt, and Bf values were considered for analysis.

##### Hemodynamic Analysis

Systolic arterial pressure, DAP, MAP, HR, CO, SV, and TPR values were averaged over the whole MI training sessions, each series (4 × 2 min30) and each resting period (3 × 1 min30). Similarly, CTRL conditions were split into seven parts, reproducing times of MI training sessions and the corresponding averaged SAP, DAP, MAP, HR, CO, SV, and TPR values were considered for analysis.

##### Autonomic Nervous System Activity (Heart Rate Variability and Cardiac Baroreflex Sensitivity Analyses)

All the IBI, SAP, and DAP values were filtered out by means of a moderate error correction filter and were edited initially by visual inspection to exclude all the undesirable beats (i.e., to ensure that each analysis for the segment was free of movement artifact and/or sharp transient change in the signal due to premature beats) which counted for <1% in every participant. 256 stable heart cycles were used for each analysis (i.e., each subject, each condition, and each posture).

For the time domain, the root mean square of successive RR interval differences (RMSSD), an indicator of parasympathetic activity, was calculated. Spectrum analysis was performed to quantify the power of spectral components in the low frequencies (LF) (0.04–0.15 Hz) and high frequencies (HF) (0.15–0.50 Hz). The very LF (0–0.04 Hz) were not addressed in the present study. However, the VLF were calculated in order to obtain the HF and LF in normalized units (nu) {HF(nu) = HF(ms^2^)/[(total power(ms^2^)-VLF(ms^2^)]; LF(nu) = LF(ms^2^)/[(total power (ms^2^)-VLF(ms^2^)]}. HF power is almost entirely mediated by the parasympathetic activity to the sinus node directly associated with respiratory activity (Pomeranz et al., 1985). Finally, the LF/HF ratio was calculated as an indicator of sympathetic over parasympathetic balance (Pagani et al., 1986; Malik et al., 1996) even if this interpretation should be viewed with caution (Reyes del Paso et al., 2013; Shaffer et al., 2014). Indeed, it is often assumed that LF/HF ratio reflects the sympathetic-parasympathetic balance (Taylor, 2006). However, it is frequently shifted due to reductions in LF power which do not reflect sympathetic nervous system activity at rest. Moreover, both branches of the ANS can be simultaneously active (Berntson and Cacioppo, 1999). Furthermore, the interactions between parasympathetic and sympathetic nervous systems are complex, non-linear, and frequently non-reciprocal (Billman, 2013).

The respiratory rate was not controlled. However, on an individual basis, we systematically checked that the respiratory sinus arrhythmia peak fell within the HF band. All recordings were consistent in this regard.

With arterial blood pressure variability (ABPV) spectral analysis, it has been shown that the LF ABPV region could be due to an endogenous neural oscillator acting on the vasculature and the hypothesis of a resonance in the baroreflex loop (Castiglioni et al., 2007). Only LF ABPV (i.e., SAP-LF and DAP-LF) was reported here.

Beat-by-beat SAP and IBI values were used to assess cardiac baroreflex sensitivity (BRS). After having collected the IBI and SAP data, beat-by-beat series have been interpolated at 5 Hz before carrying out the spectral analyses using a cubic interpolation that acts like a filter because it smooths the transition between points. Under resting conditions, transfer function analysis of gain, phase, and coherence between spontaneous oscillations in SAP and IBI were calculated in accordance with the work of Zhang et al. (1998), i.e., 0.05–0.15 Hz for the low-frequency (LF) range. The sequence method is based on the identification of at least three consecutive beats (sequence) in which an increase (or decrease) in SAP is followed by an increase (or decrease) in the IBI (Pitzalis et al., 1998; Davies et al., 2001). Only sequences with a minimum correlation coefficient of 0.85 were accepted (Laude et al., 2004). To represent arterial pressure control in the upward and downward directions, mean gain values of positive (BRS_Seq_+) and negative (BRS_Seq_−) sequences were also computed separately.

#### Electrophysiological Data

To account for background myoelectrical activity during the recording of neuromuscular responses, the root mean square (RMS) value of EMG signals was determined with an integration time of 500 prior to the stimulus artifact. SOL, GM, and GL RMS were normalized by the corresponding M_MAX_ recorded in the same condition (MI/rest and sitting/standing). During the MI training sessions, RMS was calculated taking each of the four series as a whole (4 × 2 min30), and the three rest periods (3 × 1 min30). CTRL conditions were split into seven parts reproducing times of the MI training session.

Peak-to-peak amplitudes of myoelectrical responses at rest and during MI were measured for quantitative analysis. It can be noticed that each H-reflex, reflecting spinal excitability, is generally associated with a small M-wave (noted M_atHmax_ at rest and M_atH50_), which was also measured. Variation in this response usually reflects a shift onto the recruitment curve of the H-reflex. Stability of MatH ensure similar nerve stimulation throughout the experiment (Grosprêtre and Martin, 2012). In active condition, i.e., in presence of myoelectrical activity, M_MAX_ is followed by a reflexive response, called V-wave, which is classically used as an index of the supra-spinal descending neural drive (Grospretre and Martin, 2014). Here, V-wave was used to bring further evidence regarding the descending neural drive modulation during MI in standing condition. For each muscle, all responses were normalized to maximal M-wave evoked in the same condition. Thus, H_MAX_/M_MAX_, M_atHmax_/M_MAX_, H_50_/M_SUP_, M_atH50_/M_MAX_, and V/M_MAX_ were considered as dependent variables and compared between the groups.

#### Postural Sway

In standing conditions, participants stood on a force plate (Kistler Instrument Corp., Winterthur, Switzerland). The force plate allowed continuous recording of CoP displacements in the mediolateral and antero-posterior axes. Total sway path and amplitudes were determined separately from these CoP variables of the 15 min period of standing in both MI and standing conditions. The area of CoP displacements was also analyzed as the area of the ellipse that includes 90% of the CoP points. The total CoP length and ellipse over the 15 min periods of CTRL and MI were analyzed, as well as over the seven spited parts corresponding to the MI training session (four MI series of 2.5 min and three rest periods of 1.5 min in between). For the latter analysis, CoP length was normalized to the time of each period (in mm⋅s^–1^).

### Statistical Analyses

All data are presented as mean ± SD. Normality of data sets was tested using the Shapiro–Wilk test, and variance homogeneity was tested using Levene’s test. When data were not normally distributed, a natural logarithm transformation (Ln) was applied to obtain a normal distribution, which allowed the parametric statistical comparisons.

Regarding cardiometabolic parameters, a three-way repeated measure ANOVA was performed (time × condition × posture) for the analysis of the kinetic values and a two-way ANOVA was performed to compare mean values over the session (condition × posture). Finally, ANS variables were evaluated using a two-way ANOVA (condition × posture).

For RMS and CoP data in standing posture, the two-way repeated measures analysis of variance (ANOVA) was performed with factors “time” (the seven time periods) and condition (MI vs. CTRL). Regarding PRE–POST neuromuscular data (M-waves and H-reflexes), separate analysis was performed for CTRL and MI sessions. In CTRL session a two-way repeated measures ANOVA was performed with factors “time” (PRE vs. POST) and “posture” (sitting and standing). In MI training session, a three three-way repeated measure ANOVA was performed with the addition of the factor “MI” (REST vs. MI).

When statistical significance was identified, a Sidak *post hoc* test was used to further delineate differences between conditions or time. Possible relationships between MI-induced changes in ANS, cardiometabolic, central nervous system, and postural data were screened through Pearson’s correlations. Statistical analysis was completed using Statistica (version 8.00; StatSoft Inc., Tulsa, OK, United States). The level of statistical significance was set at *p* < 0.05.

For neurophysiological data, a separate analysis was performed for each muscle.

Relative changes (in %) from CTRL to MI conditions in each posture were calculated for each variable. Pearson correlations were performed between the relative changes of ANS, cardiometabolic, and neuromuscular parameters in each posture condition.

## Results

The data of the present study can be divided into four main domains between which the effects of MI and posture were compared, namely, cardiometabolic (V̇O_2_, EE, RQ, CO, etc.), autonomous nervous system (HRV, baroreflex etc.), central nervous system (EMG activities, H-reflexes, V-waves, etc.), and posture (CoP length, area, etc.) data.

### General Characteristics

The general characteristics of the participants are present in Table 1. The self-evaluated MI quality (5.5 ± 1.1 and 5.6 ± 1.1 on average over the whole training session for sitting and standing, respectively) did not change significantly from sitting to standing, as no main effect nor interaction has been found for factors “series of MI” and “posture” (*p* > 0.05).

**TABLE 1:** General characteristics of the participants.

Age (y)	26.3 ± 4.4
Body weight (kg)	75 ± 10.1
Height (m)	1.77 ± 0.05
BMI (kg⋅m^–2^)	23.9 ± 3
WC (cm)	83.5 ± 8.2
HC (cm)	98.4 ± 7.9
WHR	0.85 ± 0.03
FM (%)	15.9 ± 5.1
FFM (kg)	59.6 ± 6.1
Hydration (%)	61.1 ± 4.3

*Mean ± SD.*

*BMI, body mass index; WC, waist circumference; HC, hip circumference; WHR, waist-to-hip ratio; FM, fat mass; FFM, fat-free mass.*

### Continuous Measurements

#### Respiratory and Metabolic Responses

Table 2 shows the results regarding respiratory and metabolic responses.

**TABLE 2:** Cardiorespiratory and metabolic responses during control (CTRL) and motor imagery (MI) sessions.

	CTRL		MI		*p*-value; *F*-value; eta squared		
	Sitting	Standing	Sitting	Standing	Condition	Position	Interaction
VO_2_ (L⋅min^–1^)	0.32 ± 0.04	0.33 ± 0.03^a^	0.32 ± 0.05^b^	0.36 ± 0.05^aaa, bb, ccc^	0.60; 0.29; 0.05	0.0001; 31.11; 0.72	0.02; 7; 0.37
VO_2_ (mL⋅min^–1^⋅kg^–1^)	4.3 ± 0.8	4.5 ± 0.7^a^	4.3 ± 0.8^b^	4.8 ± 0.9^aaa, bb, ccc^	0.54; 0.40; 0.003	0.0002; 28.40; 0.70	0.02; 7; 0.37
EE (kcal⋅min^–1^)	1.54 ± 0.17	1.62 ± 0.17^a^	1.52 ± 0.25^b^	1.73 ± 0.26^aaa, bb, ccc^	0.56; 0.32; 0.04	0.0001; 28.30; 0.71	0.02; 7.1; 0.37
RQ	0.87 ± 0.05	0.87 ± 0.05	0.92 ± 0.1	0.91 ± 0.1	0.13; 2.61; 0.18	0.79; 0.08; 0.01	0.24; 1.55; 0.11
VE (L⋅min^–1^)	10.2 ± 1.9	11.6 ± 2.4^aaa^	10.0 ± 1.7^bbb^	12.1 ± 2.3^aaa, b, ccc^	0.47; 0.56; 0.04	0.00003; 41.62;0.78	0.03; 6.7; 0.24
Vt (L)	0.72 ± 0.19	0.77 ± 0.22	0.63 ± 0.13^a, bb^	0.80 ± 0.28^ccc^	0.21; 1.75; 0.13	0.05; 4.36; 0.31	0.02; 7.93; 0.40
Bf (breath⋅min^–1^)	15.3 ± 3.4	15.8 ± 3.2	16.9 ± 4.2	16.8 ± 4.6	0.15; 2.37; 0.16	0.59; 0.30; 0.02	0.46; 0.58; 0.05

*Mean ± SD.*

*a, aaa: significantly different from sitting during control session at p < 0.05 and p < 0.001, respectively.*

*b, bb, bbb: significantly different from standing during control session at p < 0.05, p < 0.01, and p < 0.001, respectively.*

*ccc: significantly different from sitting during MI at p < 0.001.*

*CTRL, control; MI, motor imagery; VO_2_, oxygen consumption; EE, energy expenditure; RQ, respiratory quotient; VE, ventilation; Vt, tidal volume; Bf, breathing frequency. Degree of freedom = 1.*

As there was no duration effect during CTRL sessions and no sequence effect (i.e., no significant difference between series and rest periods) during MI training sessions, the mean value during each posture for each condition (e.g., mean EE during CTRL in sitting position, mean EE during CTRL in standing position, etc.) is considered for the following analyses.

There was no condition effect regarding V̇O_2_ (absolute and relative to body weight) and EE. Overall, standing posture induced significantly higher V̇O_2_ (absolute and relative to body weight) and EE values than sitting posture (*p* < 0.001). During CTRL session, sitting V̇O_2_ (absolute and relative to body weight) and EE values were significantly lower than during standing (*p* < 0.05). There was no difference in V̇O_2_ (absolute and relative to body weight) and EE between sitting CTRL and sitting MI. Standing during CTRL session induced significantly lower V̇O_2_ (absolute and relative to body weight) and EE values than during standing during MI (*p* < 0.01) and induced significantly higher values compared with sitting during MI (*p* < 0.05). MI while standing led to higher V̇O_2_ (absolute and relative to body weight) and EE values than MI during sitting (*p* < 0.001).

There was no condition effect regarding V̇E. Overall, standing posture induced significantly higher V̇E values than sitting posture (*p* < 0.001). V̇E values while sitting during a CTRL session were significantly lower than standing during CTRL and MI sessions (*p* < 0.001). There was no difference in V̇E between sitting CTRL and sitting MI. Standing during CTRL session induced significantly lower V̇E values than during standing during MI (*p* < 0.05) and significantly higher values compared with sitting during MI (*p* < 0.001). Standing led to increased V̇E values when compared with sitting during MI sessions (*p* < 0.001).

There was no conditional effect regarding Vt but there was a significant interaction. Overall, standing posture induced significantly higher Vt values than sitting posture (*p* < 0.05). Vt values while sitting during CTRL session were not significantly different than standing during CTRL and MI sessions. Sitting during CTRL session induced significantly higher Vt values than sitting during MI session (*p* < 0.05). Standing during CTRL session induced significantly higher values of Vt compared with sitting during MI (*p* < 0.01) while no difference was observed with standing during MI session. Standing led to increased Vt values when compared with sitting during MI sessions (*p* < 0.001).

No condition, posture, and interaction effects were observed for RQ and Bf.

#### Hemodynamic Responses

Table 3 presents the results regarding hemodynamic responses. Similar to the respiratory and metabolic parameters, as there was no time effect during CTRL sessions and no sequence effect (i.e., series and rest periods) during MI sessions, the mean value during each posture for each condition is presented. There was no condition and interaction effect regarding Ln-SAP, Ln-DAP, and Ln-MAP. Overall, standing position decreased Ln-SAP (*p* < 0.001), Ln DAP (*p* < 0.05), and Ln-MAP (*p* < 0.001) values compared with sitting position. Overall, CTRL sessions induced significantly higher Ln-HR and Ln-SV values than MI sessions (*p* < 0.01 and *p* < 0.001, respectively). Overall, the standing position led to increased Ln-HR and decreased Ln-SV values compared with the sitting position (*p* < 0.001). Ln-CO was significantly lower in the standing compared to the sitting position (*p* < 0.01). Ln-CO was significantly decreased during MI session in the standing position compared to other conditions (*p* < 0.05). There was no posture, condition, and interaction effect regarding Ln-TPR.

**TABLE 3:** Hemodynamic responses during CTRL and MI sessions.

	CTRL		MI		*p*-value; *F*-value; eta squared		
	Sitting	Standing	Sitting	Standing	Condition	Position	Interaction
SAP (mmHg)	125. ± 16.3	119.1 ± 14.5	126.1 ± 23.0	112.6 ± 26.9	0.48; 0.54; 0.05	0.0002; 30.21; 0.70	0.29; 1.23; 0.10
DAP (mmHg	61.6 ± 9.0	60.7 ± 6.6	64.9 ± 15.8	60.1 ± 16.2	0.94; 0.006; 0.0006	0.03; 7.4; 0.27	0.32; 1.10; 0.09
MAP (mmHg)	82.9 ± 10.7	78.7 ± 8.5	84.0 ± 17.9	74.8 ± 19.8	0.35; 0.97; 0.08	0.0002; 27.2; 0.68	0.28; 1.28; 0.10
HR (bpm)	67.2 ± 9.8	78.2 ± 14.3	62.9 ± 9.0	77.3 ± 15.5	0.002; 13.18; 0.42	0.0001; 32.38; 0.75	0.11; 3.0; 0.22
CO (L⋅min^–1^)	5.7 ± 1.1*^a^*	5.4 ± 0.8^a^	5.4 ± 0.6^a^	4.9 ± 1.2	0.0003; 29.15; 0.71	0.0001; 35.42; 0.78	0.03; 6.80; 0.25
SV (ml)	92.9 ± 17.5	73.4 ± 15.8	84.4 ± 9.9	63.2 ± 20.7	0.0002; 30.28; 0.72	0.0001; 34.36; 0.76	0.30; 1.17; 0.10
TPR (mmHg⋅s/mL)	0.96 ± 0.28	0.92 ± 0.29	0.93 ± 0.24	0.95 ± 0.21	0.81; 0.06; 0.005	0.31; 1.14; 0.09	0.55; 0.38; 0.03

*Data are presented as Mean ± SD.*

*a: significantly different from standing during MI at p < 0.05.*

*CTRL, control; MI, motor imagery; SAP, systolic arterial blood pressure; DAP, diastolic arterial blood pressure; MAP, mean blood arterial pressure; HR, heart rate; CO, cardiac output; SV, stroke volume; TPR, total peripheral resistance.*

*Degree of freedom = 1.*

#### Autonomic Nervous System: Heart Rate Variability and Cardiac Baroreflex Sensitivity Analyses

Except for Ln-LF (ms^2^), coherence-LF, and BRS seq+, all the variables showed a position effect. Indeed, IBI, RMSSD, Ln-HF (ms^2^), Ln-HF (nu), gain-LF, phase-LF, and BRS seq− were significantly higher in the sitting than the standing position (Table 4). On the contrary, Ln-LF (nu), Ln-LF/HF, Ln-SAP-LF, Ln-DAP-LF, and the number of positive and negative sequences were significantly higher in the standing than the sitting position (Table 4). IBI was significantly higher during MI compared with the CTRL condition and Ln-DAP-LF was significantly higher during CTRL compared with MI and there was no other condition or interaction effect.

**TABLE 4:** Autonomic nervous system responses during CTRL and MI sessions.

	CTRL		MI		*p*-value; *F*-value; eta squared		
	Sitting	Standing	Sitting	Standing	Condition	Position	Interaction
**Heart rate and arterial blood pressure variability**							
IBI (ms)	884 ± 120	759 ± 124	935 ± 92	775 ± 521	0.05; 4.80; 0.30	0.000003; 72.95; 0.87	0.79; 1.03; 0.07
RMSSD (ms)	53.1 ± 18.1	35.5 ± 23.6	60.6 ± 20.7	37.8 ± 24.3	0.24; 1.51; 0.12	0.02; 8.70; 0.44	0.33; 1.03; 0.09
HF (ms^2^)	1063.82 ± 765.77	506.05 ± 676.59	1156.93 ± 835.42	625.76 ± 855.51	0.92; 0.01; 0.001	0.02;7.22; 0.40	0.68; 0.18; 0.02
LF (ms^2^)	2438.56 ± 2323.49	1817.42 ± 1027.40	3254.77 ± 2050.10	2356.50 ± 1494.90	0.50; 0.48; 0.04	0.55; 0.46; 0.06	0.38; 0.84; 0.07
HF (nu)	0.35 ± 0.19	0.22 ± 0.16	0.27 ± 0.15	0.18 ± 0.12	0.26; 1.35; 0.10	0.05; 4.79; 0.31	0.81; 0.09; 0;01
LF (nu)	0.65 ± 0.19	0.78 ± 0.16	0.73 ± 0.15	0.82 ± 0.12	0.27; 1.64; 0.10	0.05; 4.78; 0.30	0.79; 0.08; 0.01
LF/HF	2.92 ± 2.42	5.47 ± 4.84	4.45 ± 3.9	7.14 ± 4.71	0.16; 2.24; 0.17	0.004; 13.61; 0.55	0.97; 0.001; 0.0001
SAP-LF (ms^2^)	33.71 ± 29.03	73.87 ± 141.53	28.10 ± 19.96	35.88 ± 22.40	0.84; 0.05; 0.004	0.04; 5.65; 0.34	0.74; 0.11; 0.01
DAP-LF (ms^2^)	8.85 ± 6.26	26.53 ± 53.16	10.03 ± 6.39	14.88 ± 8.51	0.05; 4.90; 0.38	0.001; 17.70; 0.62	0.57; 0.34; 0.57
**Cardiac Baroreflex: Transfer function analysis**							
Gain-LF (ms × mmHg^–1^)	8.0 ± 3.8	6.9 ± 4.7	9.2 ± 3.1	5.8 ± 2.3	0.65; 0.22; 0.02	0.0006; 22.75; 0.67	0.54; 0.39; 0.03
Phase-LF (rads)	0.008 ± 0.27	−0.17 ± 0.37	0.17 ± 0.21	−0.16 ± 0.22	0.57; 0.34; 0.03	0.008; 0.56; 0.49	0.11; 2.25; 0.26
Coherence-LF	0.60 ± 0.19	0.55 ± 0.23	0.57 ± 0.11	0.63 ± 0.26	0.74; 0.11; 0.01	0.21; 1.80; 0.14	0.99; 0.0001; 0.00001
**Cardiac Baroreflex: Sequence Method**							
n seq+	25.6 ± 24.0	35.1 ± 23.0	28.3 ± 17.7	48.8 ± 26.0	0.27; 1.36; 0.11	0.04; 5.35; 0.33	0.20; 1.87; 0.14
n seq−	29.4 ± 31.1	45.8 ± 33.0	30.4 ± 19.7	59.6 ± 26.0	0.53; 0.42; 0.04	0.004; 13.23; 0.55	0.21; 1.81; 0.14
BRS seq+ (ms × mmHg^–1^)	48.2 ± 100.3	8.6 ± 4.3	13.3 ± 4.5	15.4 ± 17.6	0.86; 0.03; 0.003	0.09; 3.65; 0.25	0.18; 2.02; 0.16
BRS seq− (ms × mmHg^–1^)	7.7 ± 5.0	5.2 ± 3.0	8.7 ± 3.2	6.1 ± 2.5	0.49; 0.52; 0.05	0.0001; 35.24; 0.76	0.73; 0.13; 0.01

*Data are presented as Mean ± SD. CTRL, control; MI, motor imagery; IBI, interbeat interval; RMSSD, square root of the sum of successive differences between adjacent normal R–R intervals squared; Ln, logarithm transformation; HF, high frequency; LF, low frequency; DFA, detrended fluctuation analysis; BRS, baroreflex sensitivity; n seq+ and n seq−, number of positive and negative BRS sequences, respectively; BRS seq+ and BRS seq−, mean gain values of positive and negative BRS sequences, respectively. Degree of freedom = 1.*

#### Myoelectrical Activities

**FIGURE 2:**
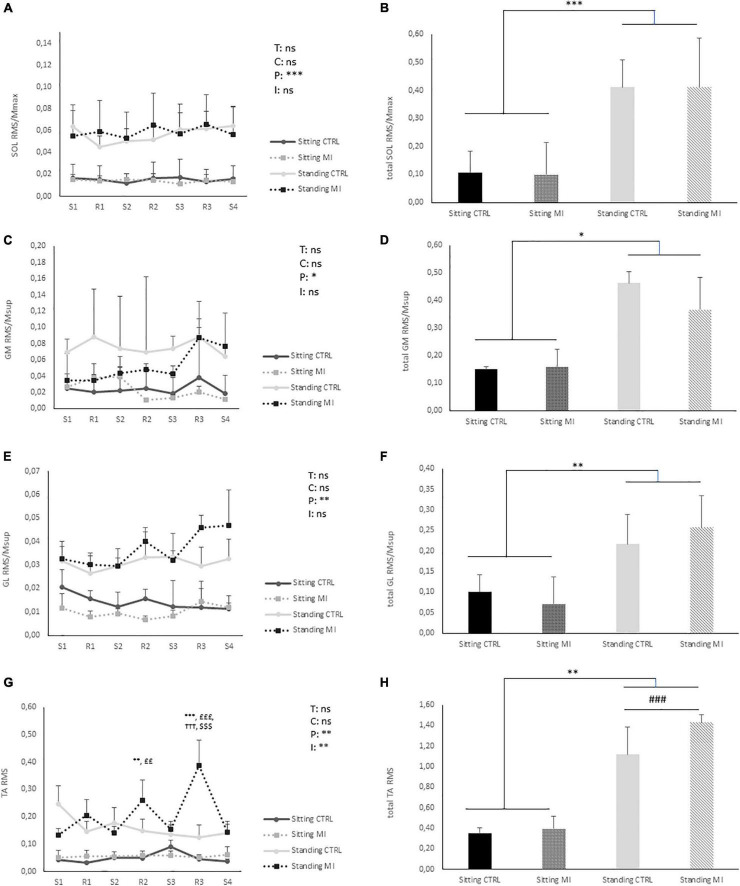
Myoelectrical recordings of leg muscles in standing and sitting postures. The root mean square (RMS) of electromyographic (EMG) activities were calculated for soleus (SOL), Gastrocnemius Medialis (GM), Gastrocnemius Lateralis (GL), and Tibialis Anterior (TA) in MI conditions (right panels) and in CTRL conditions (left panel). For SOL, GM, and GL, RMS is normalized by the maximal M-wave recorded in corresponding condition at PRE (RMS/M_MAX_). The RMS of the EMG recorded continuously during the 15-min conditions was spitted in seven parts: during the four series of MI (S1, S2, S3, and S4) and during the three rest periods (R1, R2, and R3). In the CTRL conditions the same cutting was applied. **(A)** Kinetic of SOL RMS/M_MAX_. **(B)** SOL RMS/M_MAX_ value of the entire session. **(C)** Kinetic of GM RMS/M_MAX_. **(D)** GM RMS/M_MAX_ value of the entire session. **(E)** Kinetic of GL RMS/M_MAX_. **(F)** GL RMS/M_MAX_ value of the entire session. **(G)** Kinetic of TA RMS. **(H)** TA RMS value of the entire session. T: time effect; C: condition effect; P: position effect; I: interaction effect between “time,” “condition,” and “posture”; ns: non-significant. ###: significantly different from sitting at *p* < 0.001. *, ^**^, ^***^: significantly different from sitting CTRL at *p* < 0.05, *p* < 0.01, and *p* < 0.001, respectively. **†††**: significantly different from standing CTRL at *p* < 0.001. ££, £££: significantly different from sitting MI at *p* < 0.01 and *p* < 0.001, respectively. $$$: significantly different from other points of standing MI at *p* < 0.001.

A significant effect of “posture” was found on RMS/M_MAX_ for SOL, GM, and GL (*p* < 0.001, *p* < 0.01, and *p* < 0.01, respectively) values being higher in standing as compared to sitting posture during the whole 15 min (Figure 2). There was no other main effect (time or condition) or interaction along the 15 min. However, a significant “time” × “posture” × “condition” interaction has been found for TA RMS. In MI standing condition, TA myoelectrical activity during the second and third rest period was significantly higher as compared to the rest of the condition, and significantly higher to the three other conditions (CTRL standing, MI, and CTRL sitting) (Figure 2). Overall, for total RMS activities, only a main effect of posture has been found for each of the four tested muscles, MI and CTRL not being statistically different in standing and sitting positions.

### Postural Sway

**FIGURE 3:**
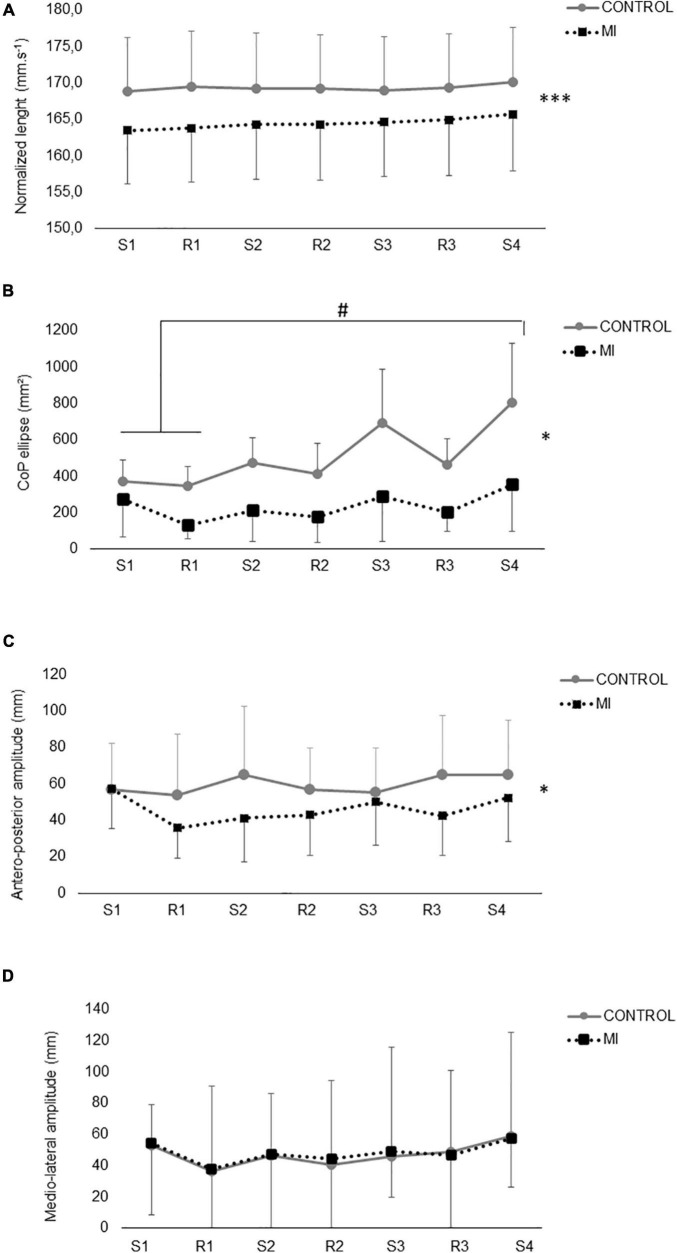
Postural sway during motor imagery (MI) and control (CTRL) in standing condition. In panels **(A–D)**, data are split according to the different phases of the motor imagery session (MI, black squares): S1, S2, S3, and S4 are for the four different successive series of MI (2.5 min), while R1, R2, and R3 represent the three rest periods between the series (1.5 min). The same cut has been performed in the CTRL session to provide valuable comparison with MI session. In panel **(A)**, length of the Center of Pressure (CoP) is normalized by the duration of each period (in mm/sec). In panel **(B)**, CoP area (ellipse) is expressed in mm^2^ in each phase. In panels **(C,D)**, the mean amplitude of the CoP displacement over the different time periods on the antero-posterior **(C)** and mediolateral **(D)** axis. * and ^***^: main effect of the factor condition (MI vs. CTRL) at *p* < 0.05 and *p* < 0.001, respectively. #: significant differences between the indicated time points, at *p* < 0.05.

A main effect of condition (*p* < 0.001) has been found for normalized CoP length, with no effect of time or interaction (Figure 3A). The total CoP length was significantly lower (*p* < 0.05) in standing MI as compared to CTRL. A significant main effect of condition, and time × condition interaction has been found for CoP ellipse area (*p* < 0.05). Over the two conditions, CoP ellipse showed a significant increase between the last time period and the first two in CTRL condition, while remaining unchanged for the MI condition (Figure 3B). A significant main effect of condition (*p* < 0.05) has been found for CoP antero-posterior amplitude, CTRL being greater than MI, with no time effect or interaction (Figure 3C). No significant main effect or interaction have been found for mediolateral CoP amplitude (Figure 3D).

### Pre-to-Post Neuromuscular Assessment

No significant main effect nor interaction have been found for maximal and submaximal M-wave in the different postures and conditions (*p* > 0.05). For all tested muscles, M_MAX_ and MatH/M_MAX_ were neither altered by MI nor by the changes from the sitting to standing position showing, respectively, (1) no changes at the level of the neuromuscular junction and (2) no changes in nerve stimulation conditions. As during the MI sessions, the background level of EMG activity measured before each PRE and POST stimulus artifact did not reveal any alteration by MI neither in sitting nor in standing posture.

**FIGURE 4:**
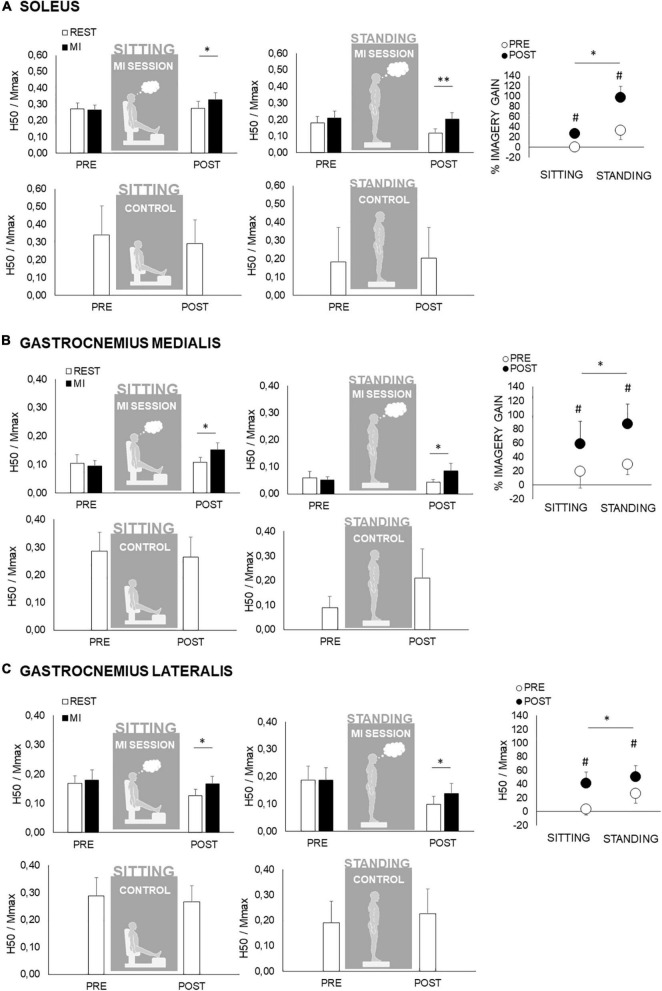
Spinal excitabilities of the triceps surae during the motor imagery (MI) and control (CTRL) conditions in the sitting and standing postures. In panels **(A–C)** are depicted responses for soleus, gastrocnemius medialis and gastrocnemius lateralis, respectively. PRE and POST responses are recorded during rest (white bars) and during MI (black bars) in sitting (left panel) and in standing condition (middle panel). In panels **(A–C)** upper panels depict data of the motor imagery session and lower panels depict data of the CTRL session. Spinal excitabilities are measured by the H-reflex recorded at 50% of its maximal value (H_50_). This response is normalized by the maximal muscle compound action potential (H_50_/M_MAX_) recorded in the same condition (MI or rest). The right panel represent the gain associated to motor imagery in the mental session {determined by the formula [(MI-REST)/REST] × 100} in each condition from PRE (white circles) to POST (black circles). This gain is calculated according to the results obtained in H-reflexes in MI and at rest, depicted in the left panels. *, ^**^: significant differences at *p* < 0.05 and *p* < 0.01, respectively. #: significant pre–post differences at *p* < 0.05 for the relative gains associated to MI.

Regarding spinal excitability, no main effect nor interaction has been found for maximal rest normalized H-reflex (H_MAX_/M_MAX_) of SOL, GM, and GL for factor “time” (PRE to POST) and condition (MI to REST). However, H_MAX_/M_MAX_ were significantly lower in standing as compared to sitting condition for SOL and GM, a main effect of posture being noted (*p* < 0.01). Regarding submaximal H-reflex (H_50_/M_MAX_), condition × time interaction has been found for each muscle (*p* < 0.05). In sitting as in standing posture, H_50_/M_MAX_ was greater during MI than during REST only at POST, while not different at PRE (Figure 4). To further this analysis, when comparing the relative changes in H_50_/M_MAX_ with MI, the gains were higher following MI practice in standing posture as compared to sitting posture (Figure 4).

In standing posture, supraspinal modulations were accounted by the concomitant analysis of V-wave accompanying maximal M-wave. In gastrocnemii, no significant main effect or interaction have been found for V/M_MAX_, being unaltered by MI in PRE as in POST condition. In contrast, soleus V/M_MAX_ exhibited a significant gain (*p* < 0.05) by MI in POST, while unchanged in PRE.

### Relationships Between the Variables

First, no significant correlation was found between relative changes from CTRL to MI of each variable in the sitting condition. Second, in the standing posture, only the relative changes from CTRL to MI of CO and those of neuromuscular parameters were significantly correlated. Indeed, a significant negative correlation was found between the relative change in SOLEUS H-reflex and the relative change in CO (*r* = −0.63, *p* < 0.05). Participants with the highest increase in H-reflex due to MI were those with the greater decrease in CO. On the contrary, relative changes of CO due to MI were positively correlated with relative changes of SOL V/M_*SUP*_ in standing posture (*r* = 0.62, *p* < 0.05). A significant correlation (*r* = 0.65, *p* < 0.05) was found between the relative change in CO due to MI and the relative change in CoP area due to MI. In other terms, the greater the decrease of the CoP induced by MI, the greater the CO was decreased by MI. Finally, both the relative changes in SOL H-reflexes and V-waves were significantly and negatively correlated with the change in CoP length (respectively *r* = −0.97 and *r* = −0.706, *p* < 0.01). Particularly, the more MI decrease CoP, the more it increases neuromuscular responses.

## Discussion

The aim of the present study was to assess neuromuscular, ANS, and cardiometabolic changes associated with acute bout of MI practice, specifically in sitting vs. standing condition. While MI did not reveal any effect on ANS, the standing posture increased the indexes of the sympathetic system activity and decreased those of the parasympathetic system activity. Moreover, MI during standing exceeds greater spinal excitability compared with the sitting posture, which was accompanied with higher V̇O_2_, EE, V̇E, and lower CO. Moreover, MI during standing induced greater spinal excitability changes as compared with the sitting posture, as well as significant changes in CoP characteristics as compared to the CTRL condition.

### Motor Imagery and the Nervous System Activation: Autonomic or Solely Voluntary?

The novelty of the present study lies in the assessment of the MI effects on different physiological systems (ANS, cardiometabolic, and neuromuscular) by varying the posture from a relaxed and low energy consuming, i.e., sitting, to a more demanding, albeit automatic, task, the standing posture.

The ANS is involved in voluntary muscle contractions through the activation of sympathetic and parasympathetic systems, but also through the stimulation of mechanoreceptors, chemoreceptors, and baroreceptors (Friedman et al., 1992; Iellamo et al., 1997). It has been shown that muscle sympathetic nerve activity and heart rate were increased proportionally with the elevation of tension during submaximal sustained handgrips (Saito et al., 1986). However, Seals (1993) noted that exceeding a minimal force, the sympathetic outflows seemed independent of the level of tension during a sustained isometric contraction, suggesting a decoupling relationship between ANS and voluntary contraction. However, these results were observed with a specific exercise modality (i.e., isometric), and Katayama and Saito (2019) recently reported that changes in muscle sympathetic nerve activity are a function of exercise modality and thus muscle contraction characteristics and position.

Specifically, regarding the effect of posture, in the present study, the postural change from sitting to standing increased the indexes of the sympathetic system activity and decreased those of the parasympathetic system activity and resulted in an increase in HR and a decrease in SAP, DAP, MAP, CO, and SV. These changes in response to postural change are commonly observed in the literature (Frey et al., 1994). Yet, significant relationships between neuromuscular and hemodynamic variables impacted by MI could only be found in standing posture, demonstrating that it is particularly prone to MI-induced modulations.

As MI shares neural circuits with motor execution, one might expect ANS to be activated during MI. Oishi et al. (1994) investigated the influence of MI on H-reflex and HR in elite speed skating athletes. During MI, which consisted of imaged sprint competition with subjects in the supine position, the authors reported an increase in cardiorespiratory parameters (i.e., HR, Bf) and a reduction of the spinal excitability. Conversely, Bunno et al. (2015) observed a greater spinal excitability and index of the cardiac sympathovagal balance (LF/HF ratio) during imaged thenar muscle contraction at 10 and 50% of maximal voluntary contraction. The difference in the characteristics of subjects and in the MI and methodology used to assess spinal excitability between these two studies may explain the discrepancies observed in terms of neuromuscular responses.

Here, the results pointed out, for the first time, that standing posture during MI magnified the ventilation, oxygen consumption, and EE responses compared to the CTRL condition. Conversely, no significant difference was observed regarding ANS activity and hemodynamic parameters, except for CO which was decreased during MI session in the standing position compared to other conditions. Interestingly, CO was the only cardiometabolic parameter associated with neuromuscular and postural variables. To our knowledge, no study investigated the relationship between cardiometabolic and neuromuscular changes with MI, and it is thus difficult to interpret the present results. However, these results may suggest that CO is associated with the regulation of balance due to the correlations with H-reflex and CoP area and the central command during MI in standing posture. The physiological pre-activation generated by the standing position during MI seems to elicit specific hemodynamic response related to neuromuscular changes. One explanation could be that MI during standing results in increased balance control that leads to a decreased blood flow.

The ANS activity is influenced by multiple external factors including, but not exhaustively, physical movement, body position, ingestion of food, cold exposure but also by emotional and cognitive tasks (Porges, 1995; Collet et al., 2013). Collet et al. (2013) provided a comprehensive overview of the interrelationship between ANS and MI, and they highlighted the role of ANS in anticipating cardiovascular and respiratory responses and in providing the necessary resources to cope with impending energy demands and/or a cognitive task. In this regard, previous studies reported that imaged or observed exercise, without any muscle contractions, led to increased arterial pressure or heart rate (Wang and Morgan, 1992; Oishi et al., 2000; Fusi et al., 2005). For instance, Wang and Morgan (1992) reported that internal and external imagery (resistance-imaged exercise) increased SAP to a similar pattern than the actual exercise while no difference was observed in the CTRL group. The authors highlighted that anticipation of exercise, due to the activation of areas of the brain during MI, triggered ANS activity, which in turn regulated arterial pressure. Similarly, Fusi et al. (2005) observed changes in ANS activity associated with cardiorespiratory responses during imagined walking. In the present study, apart for some cardiometabolic parameters in the standing condition, which is not usual in the literature, the ANS activity was not significantly altered by MI. Such a discrepancy with other studies could be partly attributed to methodological specificities. The approach to assessing ANS, the type of MI session (internal vs. external, duration, modality), and the characteristics of the subjects (i.e., age, sex, physical activity level, expertise in MI) likely influence the neurovegetative responses. In addition, the well-known inter-individual variability and low repeatability with respect to ANS responses could conceal potential differences. Standardization of experimental conditions is therefore crucial when investigating ANS activity, and we paid particular attention to normalization between sessions (i.e., nutritional status, room temperature and humidity, positions during the protocol). The heterogeneity of ANS responses to MI may suggest specific responder and non-responder profiles to MI highlighting the necessity to promote individual strategy for MI practice. Further studies with larger sample size are needed to confirm this hypothesis, especially because we observed in some subjects unusually high values of positive baroreflex sequence in the control condition only. Unexpectedly, high baroreflex value has been previously reported and explained by sequence that are not driven by the baroreceptor reflex (Laude et al., 2004). However, our specific study design could not help explain this observation. Finally, it should also be mentioned that both supraspinal and spinal autonomic regulation can contribute to specific changes in HRV indexes. In that sense, previous studies already showed the interest of methods that allows deciphering the specific spinal and supraspinal contribution to HRV (Introna et al., 1995). In the present protocol, despite greater spinal and supraspinal excitabilities of the voluntary motor system during MI while standing, the HRV indexes were not modified, and the results showed the non-significant effect of MI on autonomic regulation.

Overall, the results of the present work question the common belief that MI consistently modified ANS and cardiovascular activity and highlight the specific nature of the relationship between ANS activity, cardiometabolic regulation, and MI.

### The Effects of Motor Imagery on Energy Metabolism

While MI has become a common practice for health and performance over the past decades, to our knowledge, no study specifically investigated the potential effect of MI on energy metabolism.

In the present study, V̇O_2_ (absolute and relative to body weight) and EE were significantly increased during the standing sessions compared to basal values (i.e., sitting during the CTRL session) and to a greater extent during MI condition. This mean difference in EE of 13.5% between the sitting CTRL and the standing MI represented a slight increase of ∼26 kcal over 15 min (and less than 2 kcal between standing CTRL and MI) and is unlikely to be of significant importance in terms of general energy balance and weight regulation (Hill et al., 2003). One could agree with Hill et al. (2003), however, that the present results underlined, for the first time, possible modifications of V̇O_2_ and EE in response to MI session in the standing position, and 13.5% is substantial in the context of non-exercise activity thermogenesis. From the brain perspective, mechanisms may be similar to those of cognitive training effects with increased glucose consumption during MI associated with higher autonomic responses that consequently increase V̇.

Concerning substrate oxidation, no difference was observed in RQ between sessions. However, it is worth noting that important inter-individual variability exists and may have masked potential differences. In their study, Wang and Morgan (1992) did not observe any difference in RQ between internal, external imaged exercise, and CTRL session. Troubat et al. (2009) investigated the influence of a psychologically stressful situation (i.e., chess game) on substrate oxidation (Troubat et al., 2009). They observed an increase in RQ at the onset of the game and then a decrease during the game. The authors had no clear justifications to explain these results, and the condition differed from MI, but they pointed out the potential influence of psychological strain and cognitive task on substrate utilization. In addition, cognitive activity affected energy intake, and knowing the close interrelationship between energy intake and substrate oxidation (i.e., fuel storage and utilization), it could be hypothesized that cognitive effort likely alters fuel metabolism (Chaput and Tremblay, 2007; Chaput et al., 2008; Pérusse-Lachance et al., 2013). Indeed, this effect may be mediated *via* increased cortisol and glucose instability, which has already been found during mental work (Chaput et al., 2008). In this regard, MI session may induce changes in hormonal concentrations (e.g., cortisol, catecholamines), notably in relation to ANS activity and metabolism and substrate availability (i.e., carbohydrates, lipids) due to the increased cognitive demand and/or stress condition. In conclusion, although the present results on energy metabolism and MI are inconclusive, they suggest possible changes in response to MI, particularly in a standing position, and open new avenues for research on this topic.

### The Effects of Motor Imagery on Balance

In the present study, an overall decrease in postural sway was observed when MI was performed in the standing upright posture as compared to the standing CTRL condition. In this regard, results vary in the literature, with CoP oscillations were either increased or decreased by MI. It should be noted that methodologies vary extremely, as does the interpretation of such an effect. For instance, it has been argued that the effect of MI on postural sway is related to the MI ability of the participants (Lemos et al., 2015). Similarly, it has been argued that the pre-activation induced by maintaining balance while standing could facilitate information processing during MI (Stins et al., 2015). Since in the present study, all participants reported high scores on the MIQ-r questionnaire and there was no difference in MI ability from sitting to standing, no such relationship could be established.

Then, a wide variety of imagined tasks and modalities can be found in the literature. For instance, Rodrigues et al. (2010) asked healthy individuals to imagine stepping on their toes while assuming an upright posture, using different MI modalities (visual or kinesthetic). They found an increase in body sway when participants were in the kinesthetic modality, as evidenced by a greater CoP area. As for oscillations, they were greater during MI only in the antero-posterior axis. They attributed this effect to an increase in descending command emanating from motor regions during MI, targeting the motor system of plantar flexors. The same behavior was observed during CoP when participants had to imagine more dynamic actions such as cycling or jumping, still in a kinesthetic modality (Grangeon et al., 2011; Stins et al., 2015).

Although the present study also involved kinesthetic MI, the present results depict opposite trends. The first clue to explain this discrepancy is related to the specificity of the imagined task (isometric plantar flexion). Indeed, Grangeon et al. (2011) also found that MI alters postural sway during MI while standing but, interestingly, the direction of the modulation was a function of the imagined task. CoP length, antero-posterior, and mediolateral oscillations were reduced while imagining a finger movement, whereas these parameters were enhanced while imagining jumping. In addition to an MI task-dependency of the postural adjustments, it can be argued that CoP oscillations are specifically exacerbated when the imagined task involves a higher postural control (Boulton and Mitra, 2013; Lemos et al., 2015). The MI task in the present study did appear then to be perceived as balance challenging. Therefore, participants were able to focus their attention on mentally activating their triceps surae rather than maintaining balance. Consequently, in the present study, MI during standing could be comparable to a dual-task paradigm. Therefore, a clear allocation of cognitive resources during MI could lead to a higher automatization of postural control compared to quiet standing (CTRL condition). This effect was even more pronounced when the task was prolonged since CoP ellipse increased during the 15 min of quiet standing (CTRL condition), while staying low throughout the whole MI session.

Interestingly, although CoP sway can be affected by MI in the standing posture, most authors, in accordance to the present results, found no additional EMG activity of leg muscles during MI, supporting the lack of supplemental voluntary contraction of the calf muscle (Rodrigues et al., 2010; Lemos et al., 2015; Kolářová et al., 2016). However, although EMG activity was not quantitatively altered, different motor strategies were observed as, for example, strong relationships were found between variations in CoP and EMG during kinesthetic MI (Lemos et al., 2015). These authors then suggested that the small cortical outputs may have modified motoneuronal excitability to optimize the discharge rate of the motor units. In the present study, a surprising result was observed in TA muscular activity, which increased significantly during the resting period of the MI standing session. This last result corroborates the idea that motor strategies could differ between MI and rest in the absence of quantitative change in EMG activity, although it suggests a different hypothesis than increased motoneuronal excitability. An increase in TA activity during an exaggerated CoP displacement as compared to normal standing has been previously reported showing that TA contribution to maintaining balance may vary depending on the task (Johannsson et al., 2015). This change can be attributed to different agonist-antagonist strategies, with the quiet upright posture mainly controlled by the triceps surae. This behavior of the muscle TA may be the reflection of altered network activity at the spinal level, in the circuitry mediating TA-triceps surae relationship, rather than in motoneuron excitability itself, such as pre-synaptic circuitry. To this end, the analysis of neuromuscular parameters that change with MI in standing posture, such as spinal excitability, could provide some interesting clues.

### Additional Effects of Posture and Motor Imagery on the Voluntary Motor System

In general, standing requires a greater degree of neuromuscular control than sitting, especially in the triceps surae, to maintain the upright standing posture. The latter is also characterized by a fluctuation of the CoP position (Winter et al., 1998). This leads to a particular neural control of the leg muscles and especially the triceps surae. Indeed, the maintenance of upright standing, although it requires an overall activation of the calf muscles, leading to a greater EMG activity compared to sitting, is not solely a matter of muscular contraction. Indeed, the central command needs to be adjusted depending on these CoP displacements. However, it is important to note that the control of postural sway is mainly attributed to neural mechanisms located at the spinal level (Koceja et al., 1995; Tokuno et al., 2009; Baudry and Duchateau, 2012; Cattagni et al., 2014; Johannsson et al., 2015). Regarding the latter and in line with the present results, it has long been reported that the H-reflex, the most usual tool to assess spinal excitability, was in fact depressed from sitting to standing (Katz et al., 1988; Kawashima et al., 2003). In line with the present results, this downward regulation of spinal excitability can be attributed to many mechanisms, from pre- and post-synaptic inhibitory networks to a regulation of motoneuronal pool excitability itself. Notwithstanding, this specific neural adjustment was attributed to a descending control of spinal networks due to the change in posture, rather than to an increase in the background muscle activity from sitting to standing. In fact, this lowered H-reflex has been associated with a cortico-vestibular influence rather than the leg muscle activation required to maintain posture (Cattagni et al., 2014). This latter assumption is of importance since we did not observe any supplemental motor output with MI in the standing condition, as evidenced by similar triceps surae RMS between the CTRL and MI condition. This raised the fact that neither a greater motoneuronal output nor post-synaptic mechanisms could be accounted for the observed effects of MI in the standing posture. This was confirmed by the lack of change in maximal and submaximal M-waves, which are markers of changes occurring at lower levels, such as at the neuromuscular junction. In fact, many authors rather targeted spinal pre-synaptic inhibition as the potential main contributor of spinal regulation from sitting to standing, although being less involved when the standing posture was perturbed (Baudry and Duchateau, 2014; Johannsson et al., 2015).

Interestingly, it has recently been shown that MI specifically leads to a partial yet complete removal of pre-synaptic inhibition (Grosprêtre et al., 2019). In fact, pre-synaptic inhibition is mediated by specific structures, particularly by the primary afferent depolarizations interneurons which regulate the Ia-to-alpha motoneuronal synapse and are the main contributor of an H-reflex downward fluctuation (Rudomin and Schmidt, 1999). These interneurons were shown to have a lower activation threshold than the alpha motoneurons of the ventral horn of the spinal cord (Daniele and MacDermott, 2009). Therefore, such interneurons are likely more affected by a subliminally cortical output such as generated during MI. It was previously shown that MI can therefore enhance H-reflex amplitude in the presence of a pre-activation of such structures, such as during muscle stretch, independently of the initial reflex size (Grosprêtre et al., 2016). It can be argued that the potential mechanisms leading to an increased H-reflex during muscle lengthening are likely to be involved during standing. Indeed, the standing posture is regulated by the reflexive loop activation generated by the successive stretches of the triceps surae during antero-posterior sways (Cattagni et al., 2014). Therefore, the high initial level of pre-synaptic inhibition during standing as compared to sitting lead more room for MI to enhance the H-reflex amplitude. This is accounted in the present study by a globally greater effect of MI on H-reflex when participants were standing.

Additionally, when the MI was performed repetitively, such as during a full training session, we observed that the effect of MI on spinal excitability was exacerbated. Yet, H-reflex was not enhanced by MI in pre-measurements, in sitting nor in standing conditions, while showing a positive effect in post-measurements. In the sitting and relaxed condition, two potential candidates for such effect of prolonged MI practice has already been raised (Grosprêtre et al., 2019). Again, pre-synaptic inhibitory mechanisms could be one of the main processes involved, since the repetitive solicitation of the cortico-spinal network previously mentioned is likely to be highly solicited with repetitive MI activations. But it was also argued that a potential partial releasing of neurotransmitter in the cortico-motoneuronal synapse, which leads to higher alpha motoneuron sensibility to MI, could not be ruled out. However, the present study allows to decipher which of these two mechanisms could be mainly involved. Indeed, as previously mentioned, in the standing condition no supplemental triceps surae RMS activity has been observed during MI as compared to rest measurements. In the presence of an active motor state due to standing posture requirement, an extra quantity of neurotransmitter in the cortico-motoneuronal synapse due to MI would have led to a greater RMS observed during MI as compared to rest. Therefore, the second hypothesis can be ruled out here, in favor of a pre-synaptic inhibitory network modulation.

Finally, the lack of change in EMG activity during the 15 min standing CTRL, with no change in PRE–POST neuromuscular data, argues for a lack of neuromuscular fatigue. This emphasized that 15 min of bipedal standing is insufficient to affect neuromuscular parameters in the population tested (young healthy males). Also, performing MI did not induce additional fatigue in this condition, in accordance with a previously reported lack of neuromuscular fatigue induced by a full session of MI (Rozand et al., 2014), but can still involve slight changes in postural strategies.

### Practical Implications

Motor imagery is now recognized as a simple, safe, efficient, and cost-effective modality for enhancing motor rehabilitation and performance. It has thus emerged as a relevant approach for people with prolonged immobilization and/or inactivity (due to, for instance, to injury, surgery, or physical limitations) to prevent motor and cognitive decline or dysfunctions. The present study points out for the first time that the posture during MI is of great importance to potentiate its neuromuscular and cardiometabolic effects.

Furthermore, it should be highlighted that, in the present study, the MI task was an isometric contraction of the calf muscles, which represent a very simple task to mentally represent. Asking the participant to perform MI of such a simple task allows to mentally focus on the motor command that is not challenging regarding balance and may provide specific cardiometabolic responses. Therefore, this represents a dual task that can be used to reduce postural sway, contrary to the MI of more challenging tasks and could thus be performed by athletes but also patients with physiological limitations. The present results should be put in perspective regarding more complex mental tasks, such as MI of walking. In addition, whether these findings are consistent amongst athletes and patients warrant further study.

## Conclusion

Motor imagery during standing induced greater spinal excitability compared to the sitting posture and was accompanied with greater oxygen consumption, EE, ventilation, and lower CO. MI during standing induced specific neuromuscular and cardiometabolic changes compared to sitting, but without the expected effect on the ANS. Investigating deeper in detail, the neuronal networks involved, through H-reflex conditioning paradigms, for instance, may also allow researchers to endorse the involvement of pre-synaptic circuitry during MI in a standing posture.

## Data Availability Statement

The raw data supporting the conclusions of this article will be made available by the authors, without undue reservation.

## Ethics Statement

The studies involving human participants were reviewed and approved by Comité de Protection des Personnes (CPP) 2016-A00511-50. The patients/participants provided their written informed consent to participate in this study.

## Author Contributions

SG, UM, PG, GE, and LI contributed to the conception of the work and analyzed the data. SG, PG, GE, and LI performed the experiments. SG, UM, PG, GE, LM, and LI interpreted the data. All authors contributed to drafting the work and revising it critically.

## Conflict of Interest

The authors declare that the research was conducted in the absence of any commercial or financial relationships that could be construed as a potential conflict of interest.

## Publisher’s Note

All claims expressed in this article are solely those of the authors and do not necessarily represent those of their affiliated organizations, or those of the publisher, the editors and the reviewers. Any product that may be evaluated in this article, or claim that may be made by its manufacturer, is not guaranteed or endorsed by the publisher.

## Abbreviations

ABPV: arterial blood pressure variability
ANS: autonomic nervous system
Bf: breathing frequency
CTRL: control
CO: cardiac output
CoP: Center of Pressure
DAP: diastolic arterial pressure
EE: energy expenditure
EMG: electromyography
GL: gastrocnemius lateralis
GM: gastrocnemius medialis
HF: high frequencies
H_MAX_: maximal Hoffmann (H) reflex
HR: heart rate
H_50_: H-reflex recorded at 50% of maximal H-wave amplitude
LF: low frequencies
MAP: mean arterial pressure
MI: motor imagery
M_MAX_: maximal muscle compound action potential
RQ: respiratory quotient
SAP: systolic arterial pressure
SOL: soleus
SV: stroke volume
TA: tibialis anterior
TPR: total peripheral resistance
VCO_2_: carbon dioxide production
VE: ventilation
VL: vastus lateralis
VO_2_: oxygen consumption
VO_2_: oxygen consumption
Vt: tidal volume.
